# Clinical Outcome of Multiple Platelet-Rich Plasma Injection and Correlation with PDGF-BB in the Treatment of Knee Osteoarthritis

**DOI:** 10.3390/jpm14020183

**Published:** 2024-02-07

**Authors:** Radiyati Umi Partan, Khoirun Mukhsinin Putra, Hafizzanovian Hafizzanovian, Surya Darma, Muhammad Reagan, Putri Muthia, Afifah Salshabila Radiandina, Eny Rahmawati

**Affiliations:** 1 Division of Rheumatology, Department of Internal Medicine, Dr. Mohammad Hoesin General Hospital, Faculty of Medicine, Universitas Sriwijaya, Palembang 30126, Indonesia; 2 Stem Cell & Regenerative Therapies—From Bench to Market MSc, Faculty of Life Science & Medicine, King’s College London, London WC2R 2LS, UK; 3 Department of Clinical Pathology, Dr. Mohammad Hoesin General Hospital, Faculty of Medicine, Universitas Sriwijaya, Palembang 30126, Indonesia

**Keywords:** multiple intra-articular injections of platelet-rich plasma, multiple intra-articular injections of hyaluronic acid, VAS, WOMAC, PDGF-BB, knee osteoarthritis

## Abstract

(1) Background: Current treatments for knee osteoarthritis (KOA), such as intra-articular corticosteroids or hyaluronic acid (HA) injections, are controversial due to their ineffectiveness in preventing disease progression. Platelet-rich plasma (PRP) has become a promising and possible treatment for KOA. It is thought to enhance articular cartilage regeneration and reduce OA-related impairment. PRP contains growth factors such as PDGF-BB, which stimulates growth and inhibits joint damage. Based on numerous studies, after a certain amount of time, it was found that multiple PRP treatments reduced pain more than a single injection. This study evaluates the efficacy of multiple PRP (m-PRP) injections compared to multiple HA (m-HA) injections for KOA treatment, focusing on their correlation with PDGF-BB levels. (2) Methods: In this single-center, open-label, randomized, comparative clinical trial, 30 KOA patients received m-PRP and m-HA injections. VAS and WOMAC were used to evaluate clinical outcomes and PDGF-BB concentrations. (3) Results: The study analysis revealed a statistically significant reduction in pain indices. In both the m-PRP and m-HA groups after 12 weeks, m-PRP showed superior results. PDGF-BB concentrations also increased, with a strong negative correlation and statistical significance using Spearman’s rho. (4) Conclusions: Multiple PRP injections are safe and associated with elevated PDGF-BB, reduced VAS and WOMAC scores, providing the potential for articular cartilage regeneration and inhibiting knee osteoarthritis progression.

## 1. Introduction

Osteoarthritis (OA) is a condition characterized by the progressive deterioration of a joint. The pathological alterations observed in osteoarthritis (OA) joints include the development of fibrillation and degradation of the articular cartilage, thickening of the subchondral bone, development of osteophytes, inflammation of the synovium (synovitis), degeneration of ligaments and menisci, and hypertrophy of the joint capsule [1,2]. Osteoarthritis can result in discomfort, impaired joint function, and perhaps even disability due to joint deterioration [3,4,5]. The current treatment options for knee osteoarthritis, including the application of intra-articular corticosteroids or hyaluronic acid (HA) injections, are a subject of controversy due to several difficulties in effectively preventing the progression of the disease [6]. Intra-articular corticosteroids show temporary benefits for knee pain and impairment while also exerting adverse effects on knee structures [7,8]. The efficacy of intra-articular hyaluronic acid (IA-HA) in managing knee osteoarthritis (OA) in these individuals has raised concerns due to the contradictory recommendations seen in current clinical practice guidelines (CPGs) [9]. Hence, there is a need for new methods of treatment and improvements in osteoarthritis therapy [10,11,12]. Platelet-rich plasma (PRP) is one of the medications that have been developed for knee osteoarthritis. PRP has the potential to enhance the articular cartilage regeneration process, thereby accelerating the healing process and mitigating the advancement of OA while also minimizing OA-related impairment [11,13,14].

PRP is obtained from autologous blood and contains many mediators and growth factors that have a role in cell proliferation, migration of stem cells, angiogenesis, and a reduction in inflammation (anti-inflammation) [11,15,16]. Platelet-derived growth factor (PDGF) is an important growth factor found in PRP that plays an essential function in stimulating growth. PDGF consists of five distinct isoforms, with PDGF-BB being one of them. Within osteoarthritis, PDGF-BB functions by suppressing the apoptosis of chondrocytes and promoting the growth of fibroblasts, thereby inducing the production of a collagen matrix. Therefore, PRP has the potential to inhibit the progression of joint damage and enhance the regeneration of articular cartilage [17,18,19,20,21].

There is a lack of established protocols for the utilization of PRP in management of knee osteoarthritis (KOA). There are several PRP variants, including differences in the amount of blood collected and injected, the number of PRP preparations centrifuged, platelet activation prior to injection, the frequency of injections, and the time between injections [22,23,24]. In addition, there are several unresolved hypotheses about the application of PRP injection treatment. The number and frequency of injections needed for KOA therapy are still unresolved. Based on the numerous studies, after a certain amount of time, it was found that multiple PRP treatments reduced pain more than a single injection [25]. Presently, several clinical trials have concentrated on the comparison between multiple platelet-rich plasma (PRP) injections (m-PRP) and multiple hyaluronic acid (HA) injections (m-HA) for the treatment of knee osteoarthritis (KOA). Nevertheless, the effectiveness and safety of m-PRP and m-HA injections are still a subject of discussion in this study [26,27]. The aim of this study is to evaluate the efficacy of multiple intra-articular injections of platelet-rich plasma (m-PRP) compared to multiple intra-articular injections of hyaluronic acid (m-HA) and correlation with PDGF-BB.

This study hypothesized that administering m-PRP would be more effective in reducing VAS and WOMAC scores compared to m-HA as a result of the release of growth factors (GFs) that may have an impact on the deteriorated cartilage.

## 2. Materials and Methods

The research was an open-label, randomized, comparative clinical trial that met the inclusion criteria and had a 1:1 allocation. The research took place at the Rheumatology Division of Dr. Mohammad Hoesin Hospital in Palembang between July and December 2022. The Ethics Committee of Dr. Mohammad Hoesin General Hospital has approved this research (142/kepkrsmh/2022). The protocol of the research has been successfully submitted to the Clinical Trials Registry (NCT05579665).

### 2.1. Patient Selection and Randomization

The recruitment of consecutive research participants adhered to the KOA screening criteria set by the American College of Rheumatology (ACR) in 1990. The investigation comprised participants of all genders, aged 30 to 60 years, who had been diagnosed with grades 2 and 3 KOA using X-ray imaging and the Kellgren and Lawrence (KL) scale. Knee effusion, intra-articular injections of glucocorticoids or others like stem cell injections within three months before the study, heart or lung diseases, hematological diseases, acute or chronic infections, other autoimmune diseases, an erythrocyte sedimentation rate (ESR) > 40 mm/h, a rheumatoid factor (RF) > 1:40, hemoglobin levels < 11 g/dL, and platelet counts < 150,000/mm^3^ were all reasons participants were not allowed to take part. Severe pain, measured using a Visual Analog Scale (VAS) score higher than seven, was explicitly not considered. Additionally, those who reported experiencing exacerbating pain after receiving the injection, persisting even after receiving analgesic medication (1000 mg of paracetamol), were ineligible to continue without being removed from the research.

A randomization procedure was employed to divide the participants into two groups in a 1:1 ratio via block randomization. Using a random number generator that can be accessed at https://stattrek.com/statistics/random-number-generator.aspx, accessed on 23 July 2022, the randomization procedure was carried out (as of 23 July 2022). A non-clinical staff member was responsible for conducting the randomization procedure.

### 2.2. The Description of PRP

**Figure 1:**
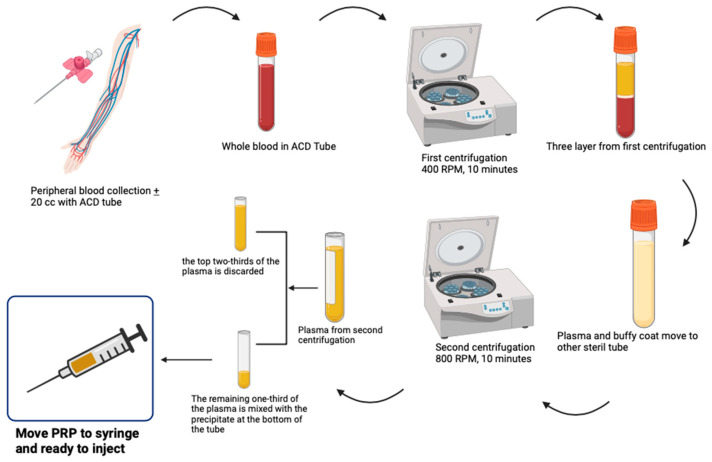
Illustrates the procedure for producing PRP with two sets of centrifuges. (This illustration was designed and produced on the online platform Biorender.com, accessed on 1 January 2024).

Autologous blood was the source of PRP (Figure 1). Peripheral venous blood + 20 mL was taken with aseptic conditions from median or antecubital vein and put into a tube with 3.2% acid citrate dextrose (ACD) anticoagulant. The blood obtained in less than 30 min was then centrifuged using a Hettich Benchtop Centrifuge Rotina 380R, manufactured by Andreas Hetich GMBH & Co. KG, Germany (2022). First, it was centrifuged at 400 RPM for 10 min, resulting in three layers (plasma, buffy coat, and sediment). The plasma and buffy coat were moved to another sterile tube, then centrifuged again with a second centrifuge at 800 RPM for 10 min. As a result of the centrifuge, the top two-thirds of the plasma were discarded, and the remaining third of the plasma mixed with the sediment at the bottom of the tube. The results of this mixing were transferred to a 2 cc syringe and then immediately injected intra-articularly into the knee. The central laboratory of Dr. Mohammad Hoesin Hospital was responsible for conducting all production procedures. The first group received weekly PRP injections given by rheumatologists for five weeks.

### 2.3. The Description of HA

The hyaluronic acid used for this clinical trial was Umaron, produced by PT. Pratapa Nirmala (FAHRENHEIT) in Indonesia. The content of each vial was 2 mL of sodium hyaluronate. The second group received weekly HA injections given by rheumatologists for five weeks.

### 2.4. Clinical Outcomes Measures

The clinical outcomes of each participant were evaluated using the Visual Analogue Scale (VAS) and the Western Ontario and McMaster University Arthritis Index (WOMAC) questionnaires. The questionnaire for WOMAC comprised 26 questions specifically crafted to measure pain, 2 questions intended to assess rigidity, and 17 questions intended to evaluate functional limitations and overall function in knee osteoarthritis patients. The VAS and WOMAC assessments were conducted before the administration of intra-articular injections and after 12 weeks. Clinical staff who were not a part of the research team evaluated VAS and WOMAC assessments.

### 2.5. Biochemical Assay

Concentrations of Platelet-Derived Growth Factor-BB (PDGF-BB) were quantified in platelet-rich plasma (PRP) samples. The quantitative assessment of PDGF-BB was performed using an enzyme-linked immunosorbent assay. The enzyme-linked immunosorbent assay kit offered by Cloud-Clone Corp. Katy, TX 77494, USA is designed to detect platelet-derived growth factor BB (PDGF-BB) in Homo sapiens (human) species. The test has a high level of sensitivity and exceptional specificity in detecting PDGF-BB, with a minimum detectable dosage generally below 0.113 ng/mL. The central laboratory of Dr. Mohammad Hoesin Hospital was responsible for conducting all procedures. The procedures were executed in accordance with the instructions provided by the manufacturers.

### 2.6. Statistical Analysis

The data analysis was conducted utilizing the IBM SPSS statistics software version 25.0 tool produced by SPSS Corp. in Chicago, IL, USA. The Kolmogorov–Smirnov and Shapiro–Wilk tests were utilized to assess the normality of the distributions. The research presented the participants’ characteristics by providing either the frequency or mean value, along with the standard deviation for data that conformed to a normal distribution. When the data exhibited a non-normal distribution, the median value was employed with the range, which encompassed the minimum and maximum values. The study utilized either the Mann–Whitney U test or the unpaired *t*-test. The pre- and post-scores of two similar groups were evaluated using either the paired *t*-test or the Wilcoxon test to see if the supplement administration resulted in a statistically significant improvement. A correlation was determined using Spearman’s rho test. A *p*-value below 0.05 was determined to be statistically significant. The research team analyzed and interpreted the data.

## 3. Results

### 3.1. Basic Characteristic

**Figure 2:**
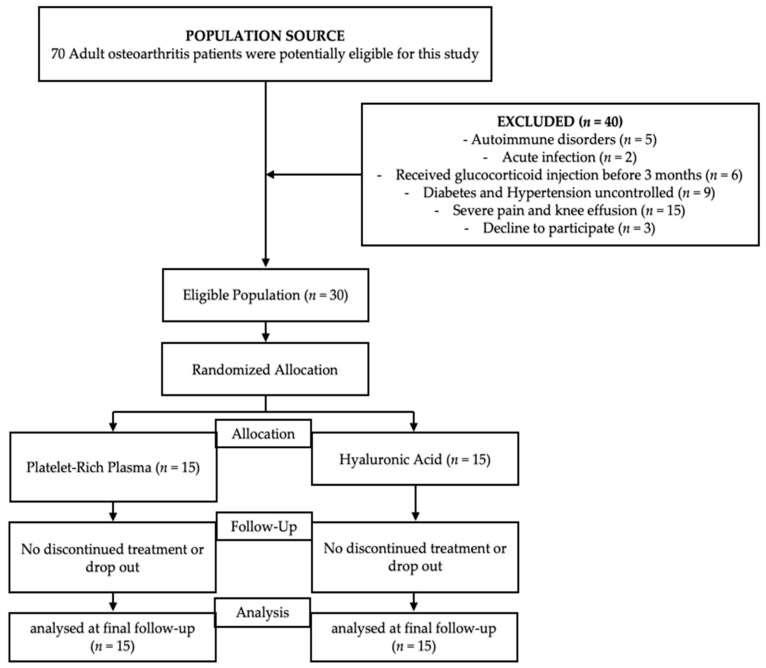
Flowchart illustrating the process of screening, randomization, and determining the sample size for analysis.

Thirty subjects met the inclusion criteria. As shown in Figure 2, every participant completed the study. From July to December 2022, participants underwent ongoing monitoring following their recruitment. All participants were randomly allocated into two groups: 15 people received PRP, while the remaining 15 received HA.

The basic features of the participants are detailed in Table 1. The results of the statistical analysis showed that there were no significant differences between the two groups in terms of age, symptom duration, gender, body mass index (BMI), Kellgren–Lawrence grade for knee osteoarthritis (KOA), physical activity goals, or serum 25(OH)D concentrations. The study primarily involved female participants, the majority of whom were diagnosed with Kellgren–Lawrence grade 3 osteoarthritis. Furthermore, they admitted to engaging in a lack of physical activity.

### 3.2. Clinical Outcomes

**Figure 3:**
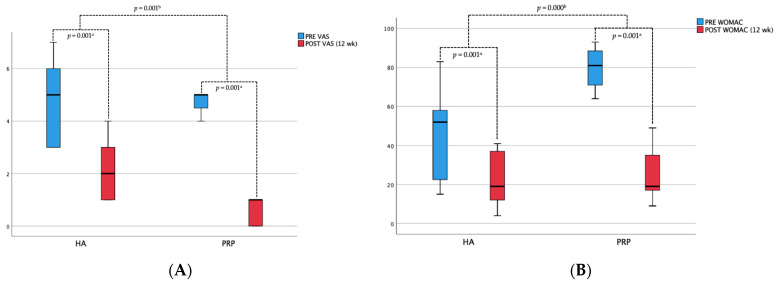
Box diagram illustrating the treatment effect of m-PRP injection and m-HA injection measurements for week 12 before and after therapy. The clinical efficacy of the treatment was evaluated using two instruments: (**A**) the Visual Analogue Scale (VAS) and; (**B**) the Osteoarthritis Index of Western Ontario and McMaster University (WOMAC). a = Wilcoxon Test; b = Mann–Whitney Test, indicating whether the difference between pre- and post-evaluations is statistically significant (*p* < 0.05).

The assessments of m-PRP injection and m-HA injection outcomes following therapy involved both subjective and objective assessments of the patients. The VAS and WOMAC assessments were compared prior to and following the 12-week treatment period, as illustrated in Figure 3. A decrease in the average pain scores was noted in both groups after 12 weeks. In the PRP groups, the mean VAS decreased significantly from 5 to 1, the reduction mean VAS before and after 12-week treatment were −4 ± 0.67 (95% CI, mean ± SD), and the mean WOMAC scores decreased significantly from 79 to 20, the reduction mean WOMAC scores before and after 12-week treatment were −59 ± 6.26 (95% CI, mean ± SD). Moreover, in the HA groups, the mean VAS decreased significantly from 5 to 2, the reduction mean VAS before and after 12-week treatment were −3 ± 1.24 (95% CI, mean ± SD), and the mean WOMAC scores decreased only from 55 to 21, the reduction mean WOMAC scores before and after 12-week treatment were −21 ± 14.07 (95% CI, mean ± SD). The statistical analysis revealed a significant difference between both groups in the mean reduction in pain intensity before and after therapy (*p* < 0.05). It is evident that the decrease shown in the PRP groups was more significant compared to the decrease in the HA group.

### 3.3. Biochemical Results

The mean platelet count in this research was 309,000/mm^3^. After the process of PRP, the mean platelet count reached 501,000/mm^3^, indicating that the approach employed in this study resulted in a 62.14% increase in platelet concentration from whole blood to PRP. After 12 weeks of observation, it was observed that platelet-rich plasma groups displayed an increase in PDGF-BB concentrations. The mean PDGF-BB concentrations showed a rise of 462.16 ± 116.19 pg/mL (95% CI, mean ± SD) after 12 weeks. The present investigation examines the possibility of a correlation between elevations in PDGF-BB levels and alterations in VAS and WOMAC scores.

**Figure 4:**
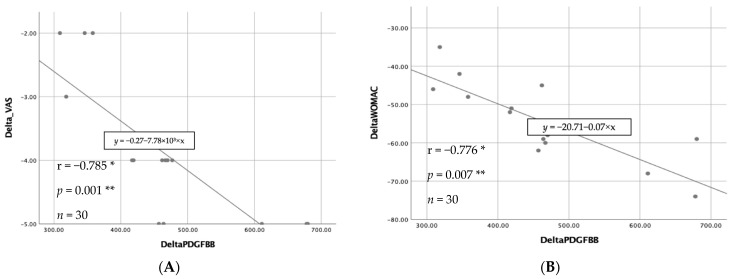
A correlation between increasing PDGF-BB levels and changes in VAS (**A**) and WOMAC scores (**B**). * Spearman’s rho test; ** *p* < 0.05. If the value of r = 0.0 to <0.2; very weak, r = 0.2 to <0.4; weak, r = 0.4 to <0.6; moderate, r = 0.6 to <0.8; strong, r = 0.8 to 1 = very strong.

The correlation between increasing PDGF-BB concentrations and changes in VAS and WOMAC scores was analyzed using Spearman’s rho. There was a strong negative correlation and statistical significance between these two variables, as shown by Figure 4.

## 4. Discussion

According to this study, administering multiple PRP and multiple HA intra-articular injections is associated with decreased pain in those suffering from knee osteoarthritis. The mean VAS in the PRP group decreased significantly from 5 to 1, an 80% reduction from the initial values, following a period of 12 weeks. On the contrary, the HA group documented a mean VAS reduction from 5 to 2, which corresponds to a decrease of 60%. During the course of the 12-week duration, substantial enhancements in WOMAC scores were observed in both groups. The mean WOMAC scores in the PRP group decreased significantly from 79 to 20, representing a 75% reduction from the baseline. On the contrary, the HA group witnessed a decline of 21 points, or approximately 61%, as opposed to the initial count of 55. According to established criteria, a pain reduction of at least 30% is indicative of significant clinically relevant changes, which means that the study had a positive outcome [28,29]. The present data suggest that m-PRP is superior to the m-HA preparation used in this study. A study involving 94 patients, discovered that multiple PRP injections intra-articular at a 6-week interval for KOA enhanced clinical outcomes from 6 weeks to 1 year. A meta-analysis of 463 patients also found that patients who received the m-PRP injections had significantly lower scores on VAS and WOMAC than patients who received the m-HA injections. A RCT involving 162 participants revealed that administering multiple PRP injections is more efficacious than single injections and hyaluronic acid in treating early osteoarthritis in the knees [25,30,31].

The reduction in pain observed in OA can be due to the suppression of joint inflammation. This process is achieved by controlling the activity of NF-κB and COX-2, which are the primary components of inflammation. Additional reasons may include the suppression of NF-κB transactivation activity through the action of HGF, a crucial cytokine found in PRP α-granules, or the anti-inflammatory effect of suppressing cell chemotaxis, especially in monocytes. The study conducted by Wu et al. showed that PRP has the ability to counteract the inflammatory processes triggered by IL-1β and TNF-α. Inhibiting the expression of the genes for IL-1β, COX-2, and MMP-2 helps achieve this. Eighty-eight Sudi, as described by Lee et al., observed elevated mRNA levels of the cannabinoid receptors CB1 and CB2, which are associated with pain relief and a reduction in inflammation. This finding suggests a potential mechanism for the analgesic benefits of PRP [32,33,34].

PRP contains an autologous concentration of human platelets in a limited volume of plasma. According to prior research, the platelet concentration in PRP necessary for therapeutic administration is 1,000,000 platelets/μL. In the case where the platelet count in whole blood is 200,000 ± 75,000/μL, PRP intended for therapeutic purposes should increase by an average of 400% from its initial value [35,36,37]. Numerous protocols were investigated in order to elevate the concentration of platelets in PRP. It is crucial to differentiate between simple and double centrifugation procedures, with the latter requiring a more extensive preparation period but yielding platelet-concentrated plasma. Platelet concentrations sufficient to induce a therapeutic effect are achieved using double centrifugation techniques in all cases [22,23,35].

This study involved doing double centrifugations at two different speeds, specifically 400 RPM and 800 RPM. The results showed a significant rise in platelet counts, specifically by 62.14%, following the centrifugation process. While the therapy outcome is still distant from the desired objective, there has been a significant alleviation in patients’ discomfort. A significant quantity of platelets has been detected in PRP. Many growth factors and other proteins are contained in platelet alpha granules (α), which can attract and stimulate stem cells to the site of platelet activation, where they can also regenerate. For therapeutic purposes, including knee osteoarthritis, PRP has been utilized to mobilize and stimulate stem cells. During the joint regeneration process, PRP can stimulate synoviocytes to make more hyaluronic acid, encourage osteoblast cell proliferation and collagen production, and reduce osteoclast activity. It can also enhance the chemotaxis and proliferation of fibroblast cells in damaged joint tissue [33,38,39,40,41].

There are growth factor proteins in the PRP. These are platelet-derived growth factors (PDGF), vascular endothelial growth factors (VEGF), transforming growth factors-β (TGFβ), and epithelial growth factors (EGF). PDGF-BB is one of the growth factors included in PRP. PDGF-BB functions by activating several signaling pathways that inhibit the process of apoptosis in chondrocytes affected by OA [42,43]. This inhibition of apoptosis leads to a decrease in the progression of OA and an enhancement in cell regeneration. Normally, the levels of PDGF-BB after platelet activation should exhibit a straightforward and proportionate correlation with the quantity of platelets. As the quantity of activated platelets rises, the release of PDGF-BB by α granules likewise increases [44,45,46]. Because of the irreversibility of the development of OA, there is a need for therapy techniques that might inhibit the degenerative process. PDGF-BB can facilitate the regeneration process of deteriorating cartilage. Based on the conducted research, the results indicate a substantial association between PDGF-BB in PRP and pain reduction. The graph showing the average VAS and WOMAC scores, which decreased after the administration of PRP injection, serves as evidence for this. Increasing PDGF-BB levels was found to have a strong negative relationship with changes in VAS and WOMAC scores (Figure 4).

**Figure 5:**
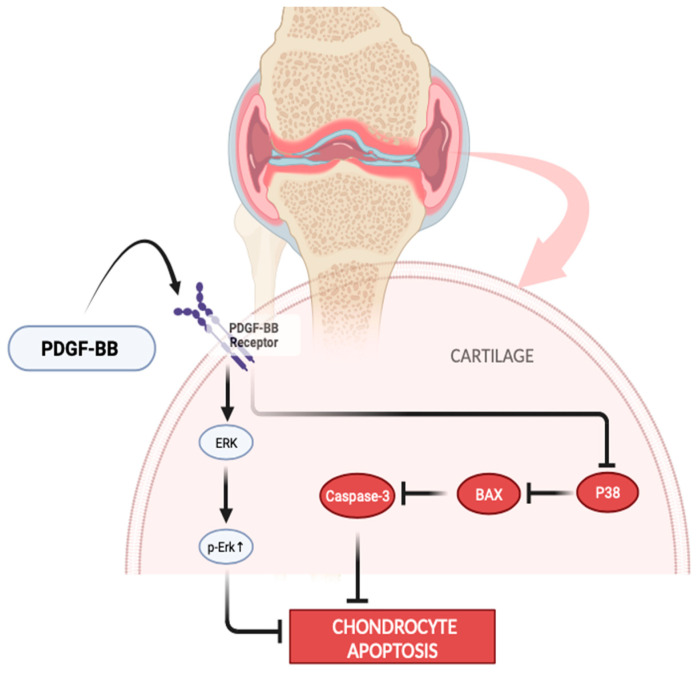
The role of PDGF-BB in condrocytes in inhibiting the progression of KOA. →: Stimulate ⊣: inhibition (This illustration was designed and generated using the web platform Biorender.com, accessed on 1 January 2024).

Figure 5 illustrates the process by which PDGF-BB may inhibit the progression of OA. Through its effects on the Erk and p38 signaling pathways, PDGF-BB can stop chondrocytes from going through apoptosis in OA. PDGF-BB induces activation of the Erk signaling cascade through its interaction with the PDGFR (Platelet-Derived Growth Factor Receptor). The phosphorylation cascade process is initiated by this binding, which eventually results in the activation of ERK. ERK activation may inhibit apoptosis by phosphorylating and deactivating proteins that promote apoptosis or by controlling the production of proteins that inhibit apoptosis [17,47,48,49]. In the P38 signaling pathway, PDGF-BB has the ability to decrease the activity of p38, resulting in a decrease in BAX protein levels. This decrease in BAX protein levels can block the release of cytochrome c from mitochondria and inhibit the activation of caspase 3. This mechanism has the ability to prevent the apoptosis of chondrocytes, thereby reducing the advancement of OA [48,50,51,52].

Several studies have shown evidence that PDGF-BB inhibits the growth of osteophytes and cartilage hypertrophy while also stimulating the healing process of cartilage and reducing inflammation. These combined actions may contribute to the protective effects of PDGF-BB in OA. Zhengchao et al. demonstrated that PDGF-BB inhibits the p38/Bax/caspase-3 pathway and upregulates Erk phosphorylation to prevent chondrocyte apoptosis in rodents. Furthermore, in a murine OA model, we discovered that PDGF-BB binding to PDGFR could stimulate chondrocyte proliferation and cartilage matrix synthesis [47,48]. PRP is a very promising biological therapy for the treatment of knee osteoarthritis. Several studies have shown effectiveness in a diverse range of difficult settings. Acquiring insight into the variables that lead to this diversity will enable physicians and researchers to effectively utilize PRP and better delineate its function in treating different clinical diseases. Some individuals experienced only minimal side effects, including moderate discomfort and mild effusion, following the injections.

The limitations of our study are the low number of subjects in each group; there was no blinding; there was no mock phlebotomy to harvest blood in the HA group. The pre-treatment groups may not have been completely comparable, as the mean pre-treatment WOMAC score was considerably greater in the PRP group. There may have been a placebo effect, as injected saline has also been shown to improve VAS and WOMAC scores [53]. The inquiry was a short follow-up, with subsequent examinations carried out exclusively within a single research facility. We were unable to evaluate the cartilage directly using high-resolution MRI or microscopic evaluation, which may have yielded more objective data for determining its effectiveness. Because of its perceived lack of effectiveness, HA is not recommended by the American College of Rheumatology or the American Academy of Orthopaedic Surgeons, although EULAR considers it an option.

Due to a lack of data on effectiveness, PRP is strongly recommended by the American College of Rheumatology [54]. PRP is only limitedly recommended by the American Academy of Orthopaedic Surgeons because of inadequate data [55]. Thus, the effectiveness of PRP for OA has not yet been established, and further research is required.

## 5. Conclusions

Multiple PRP injections may have demonstrated more efficacy in decreasing VAS and WOMAC scores compared to multiple HA injections. Furthermore, the concentration of PDGF-BB showed a considerable increase after a period of 12 weeks of observation. Based on these findings, the study suggests that PRP might be a reliable therapy option for KOA. Future research on PRP for knee osteoarthritis should involve conducting long-term follow-up and measuring additional growth factor concentrations.

## Figures and Tables

**Table 1:** Basic characteristics.

Variable	Total *n* = 30 (%)	Intervention Group	
		PRP (%)	HA (%)
Age, y, mean ± SD		55.5 ± 6.2	54.3 ± 9.1
Symptom duration, mo, mean (range)		60.4 (7–360)	64.5 (4–360)
Gender			
Female	28 (93)	14 (93)	14 (93)
Male	2 (7)	1 (7)	1 (7)
BMI			
Severe Underweight (<17)			
Underweight (17–18.4)	0	0	0
Normal (18.5–25)	3 (20)	0	3 (20)
Overweight (25.1–27)	15 (50)	9 (60)	6 (40)
Obese (>27)	12 (30)	6 (40)	6 (40)
Physical Activity			
Mild	18 (60)	12 (80)	6 (40)
Moderate	12 (40)	3 (20)	9 (60)
Severe			
Kellgren Lawrence Grade			
Grade 2	8 (27)	3 (20)	5 (33)
Grade 3	22 (73)	12 (80)	10 (67)
Serum 25(OH)D Level			
Normal			
Insufficiency	10 (33)	0	10 (67)
Deficiency	20 (67)	15 (100)	5 (33)
Mean Pre-VAS	15 (50)	5	5
Mean Pre-Womac	15 (50)	79	55
Mean Post-VAS (12 weeks)	15 (50)	1	2
Mean Post-Womac (12 weeks)	15 (50)	20	21

## Data Availability

This study lacked the creation or analysis of any new data. Data sharing is not relevant to this subject.
